# Spatially selective clearance of inflammation-mediated reactive oxygen species in injured kidney cells

**DOI:** 10.7150/thno.112905

**Published:** 2026-01-01

**Authors:** Maowei Xie, Xin Fu, Yufei Lan, Yuping Zhang, Haixin Li, Yan Su, Kun Zhou, Ying Zhang, Xuejing Ding, Jian He, Zhilei Mao

**Affiliations:** 1 Department of Nephrology, Hainan General Hospital, Hainan Affiliated Hospital of Hainan Medical University, Haikou 570311, China.; 2 Department of Oncology, Zhujiang Hospital, Southern Medical University, Guangzhou 510282, China.; 3 Department of Neurosurgery, The National Key Clinical Specialty, The Engineering Technology Research Center of Education Ministry of China on Diagnosis and Treatment of Cerebrovascular Disease, Guangdong Provincial Key Laboratory on Brain Function Repair and Regeneration, The Neurosurgery Institute of Guangdong Province, Zhujiang Hospital, Southern Medical University, Guangzhou 510282, China.; 4 Changzhou Maternity and Child Health Care Hospital, Changzhou Medical Center, Nanjing Medical University, Changzhou 213003, Jiangsu, China.; 5 Department of Epidemiology, Center for Global Health, School of Public Health, Nanjing Medical University, Nanjing 211166, China.; 6 Department of Nuclear Medicine, Nanjing Drum Tower Hospital, The Affiliated Hospital of Nanjing University Medical School, Nanjing 210008, China.

**Keywords:** acute kidney injury, tetrahedral framework nucleic acid, platelets, reactive oxygen species, renal fibrosis

## Abstract

**Rationale:** Kidney injury is characterized by the accumulation of reactive oxygen species (ROS). Current ROS scavengers utilized in the treatment of clinical kidney injury lack the capacity for spatially selective ROS scavenging within the injured cells, often resulting in irreversible damage to healthy tissues.

**Methods:** We developed a spatially selective therapeutic strategy using platelet-shipped tetrahedral framework nucleic acids (TFNAs@PLT).

**Results:** In this system, platelets achieve spatial targeting of injured kidney cells by responding to inflammatory signals and releasing TFNAs following TNF-α activation. TFNAs subsequently exert a potent antioxidant effect in the injured cells. We have demonstrated that TFNAs@PLT reduces pyroptosis and apoptosis through the Caspase-3/GSDME pathway and the NLRP3-mediated Caspase-1/IL-1β pathway in the acute kidney injury (AKI) mouse model, while also inhibiting renal fibrosis *via* the NLRP3/Caspase-1 and the TNF-α/NF-κB pathways in the chronic kidney disease (CKD) mouse model.

**Conclusion:** TFNAs@PLT offers a safe and effective therapeutic strategy for kidney injury and other inflammation-mediated diseases.

## Introduction

Acute kidney injury (AKI) and chronic kidney disease (CKD), central clinical kidney disorders with temporal progression, are characterized by the accumulation and imbalance of reactive oxygen species (ROS) 1. Therefore, antioxidant therapy is a vital adjuvant treatment for AKI and CKD 2. However, traditional clinical ROS scavengers are non-selective and lack spatial specificity. The indiscriminate use of antioxidant reagents can deplete essential ROS necessary for normal cellular physiological processes, such as autophagy 3. To overcome this issue, it is vital to construct a system with a high spatially selective ROS scavenging ability.

Tetrahedral framework nucleic acids (TFNAs) are emerging nanomedicines for renal injury repair 4, 5. They can be rapidly and efficiently synthesized through a simple process involving one hour of thermal annealing of four DNA strands. Compared to traditional clinical ROS scavengers, TFNAs possess not only the anti-inflammatory and antioxidant effects 6, but also the significant renal retention capability, enabling long-term repair of kidney damage while minimizing harm to normal tissues 7. TFNAs can penetrate biological barriers, thereby gaining better efficiency 8, 9. However, they still fail to achieve spatially selective ROS scavenging within the injured kidney. Thus, there is an urgent need to develop more sophisticated technologies with controlled release mechanisms.

Cell-cloaked nanoparticles are undergoing extensive investigation as an efficacious approach in biomimetic drug delivery systems, given their ability to target specific cells, prolong circulation time, and exhibit strong biocompatibility 10. Notably, platelet-cloaked nanomedicines are of great interest to researchers, stemming from their platelet-like characteristics, including tumor cell adhesion, pathogen interaction, and immune evasion 11, 12. As natural constituents of the bloodstream, platelets (PLT) exhibit remarkable biocompatibility. Furthermore, they respond to inflammatory signals from damaged tissues, such as interleukin-6 (IL-6), facilitating spatially selective aggregation 13. They can also be triggered by tumor necrosis factor-α (TNF-α) released from injured cells, leading to the discharge of their contents and subsequent therapeutic effects 14. However, to the best of our knowledge, the application of PLT as drug carriers for the spatially selective treatment of renal injury is still rare.

**Figure 1:**
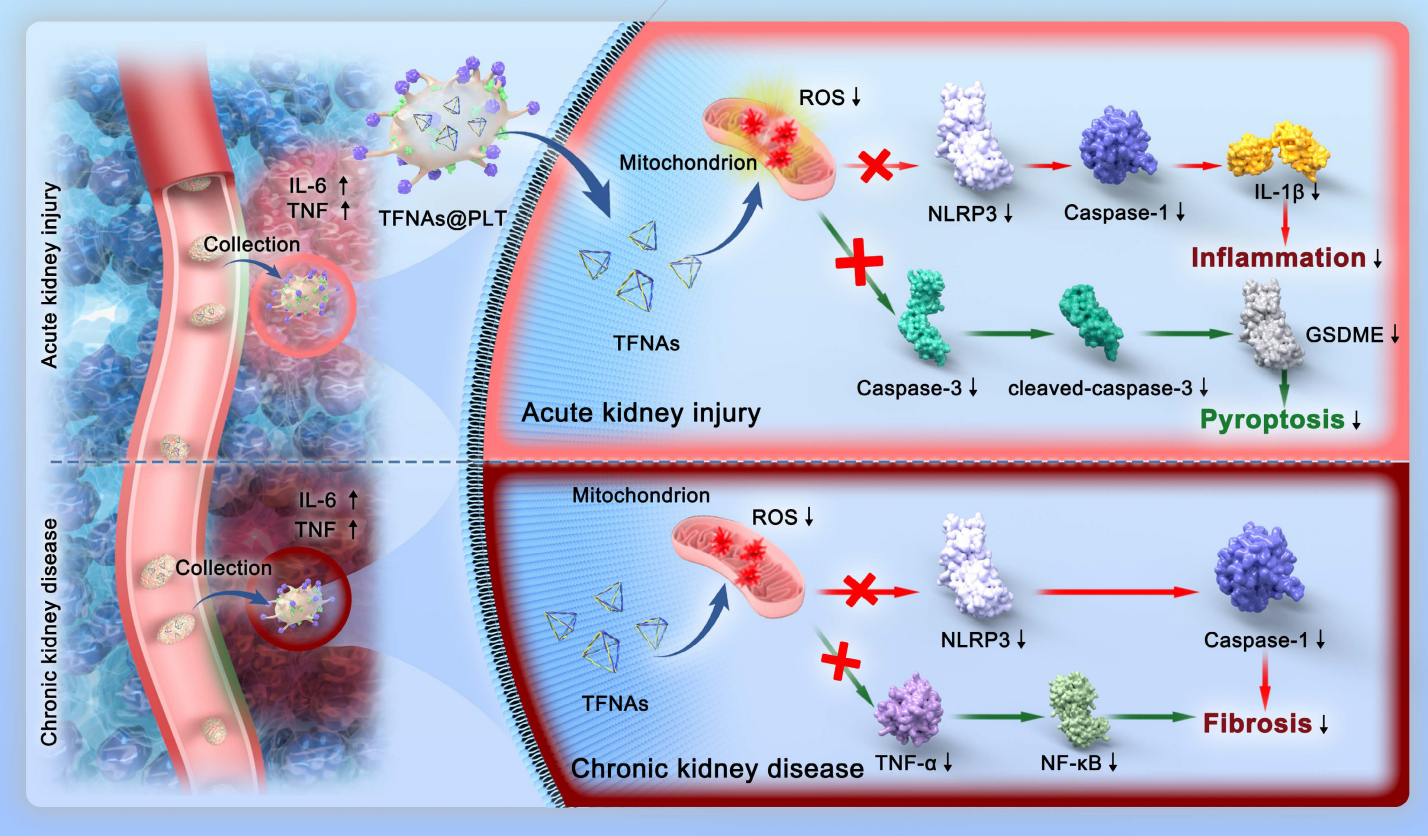
** Schematic illustration of the mechanisms for TFNAs@PLT treatment of AKI and CKD.** TFNAs@PLT were recruited by inflammatory signals and activated by TNF-α to release their contents. Subsequently, TFNAs entered the injured kidney cells, quenching ROS and inducing anti-inflammatory effects to facilitate apoptosis and pyroptosis *via* NLRP3-mediated Caspase-1/IL-1β and Caspase-3/GSDME pathways in AKI, and ameliorated renal fibrosis *via* NLRP3/Caspase-1 and TNF-α/NF-κB pathways in a CKD mouse model.

In this study, we present a strategy for achieving spatially selective treatment of renal injuries, including AKI and CKD, through the development of a platelet-cloaked nanosystem (TFNAs@PLT). The operational mechanism of this nanosystem is illustrated in Figure 1. In brief, TFNAs@PLT is prepared by the extensive encapsulation of TFNAs with PLT and selectively accumulates in the injured kidney under the influence of inflammatory factors. Subsequently, TFNAs@PLT is activated by TNF-α, releasing TFNAs to neutralize ROS at the injury site. We demonstrated that TFNAs@PLT could reduce inflammation-mediated pyroptosis and apoptosis *via* Caspase-3/GSDME and NLRP3-mediated Caspase-1/IL-1β pathways in AKI, while also alleviating renal fibrosis through NLRP3/Caspase-1 and TNF-α/NF-κB pathways in CKD. Our findings confirmed the effectiveness and safety of TFNAs@PLT in treating AKI and CKD, without causing significant adverse effects on healthy tissues. Thus, we believe our study can revolutionize the treatment landscape for kidney diseases and other inflammation-associated conditions.

## Materials and Methods

### Synthesis of TFNAs@PLT

For PLT extraction, whole blood was collected from the hearts of specific pathogen-free (SPF) Sprague-Dawley (SD) rats after administration of anesthesia, and the PLTs were isolated by gradient centrifugation. Briefly, for supernatant collection, the freshly collected rat blood was centrifuged at 200 × g (10 min) three times. To prevent PLT aggregation, we added 1 μM of prostaglandin E1 (PGE1). The samples were centrifuged at 1800 × g for 20 min to collect the pellets. TFNA synthesis was carried out according to a previous study 15, which involved the thermal annealing of a mixture of four DNA strands (S1, S2, S3, S4). The sequences of these strands (5′ to 3′) used to assemble the TFNAs are provided in Supplementary Table S1. The construction of TFNAs@PLT was performed according to previously published research 16, 17. In brief, PLTs were intermittently sonicated for 10 min with a 30 s on and 10 s off cycle (40 Hz, 80 W) using an FS30D sonicator (Fisher Scientific, China) to form tiny pores permitting the entrance of TFNAs. Then TFNAs were immediately added to the pore-prepared PLTs and incubated. After embedding, the prepared samples were transferred to a hyperfiltration tube and centrifuged for 10 min at 1000 × g to separate the unsuccessfully embedded TFNAs. Finally, the prepared TFNAs@PLT were dissolved in PBS for the following characterization assays and experiments.

### Characterization of TFNAs@PLT

The morphology observation of TFNAs@PLT and PLT was carried out using transmission electron microscopy (TEM) and scanning electron microscopy (SEM) (JSM-6700F, JEOL Co., Japan). Briefly, TFNAs@PLT and PLTs were added to glutaraldehyde solution at a ratio of 30:1 and incubated overnight for fixation. Subsequently, the samples were centrifuged, and the pellets were collected, followed by three PBS washes, and then dissolved in pure water before being dropped onto a silicon wafer. After drying, the PLTs and TFNAs@PLT were coated with gold film, and observations were carried out within 30 min. For TEM analysis, a dilute aqueous dispersion of TFNAs was drop-cast onto a carbon-coated copper TEM grid. The sample was allowed to air-dry thoroughly at room temperature to remove any solvent. The sample was imaged under high vacuum at an accelerating voltage of 120 kV to obtain the morphology. We measured the zeta potentials of PLTs, TFNAs, and TFNAs@PLT using a Zetasizer (Nano ZS90, Malvern, UK). To realize the monitoring of TFNAs@PLT, the Cy5-labeled TFNAs, as well as the FITC-labeled PLTs, were applied to form a visible TFNAs@PLT. For this, Cy5 was designed into one of the chains of TFNAs. FITC was added and incubated to stain the PLTs; then, the mixture was dialyzed in PBS for 12 h to discard the unbound FITC. The labeled TFNAs@PLT was prepared according to the same procedure as TFNAs@PLT with ultrasound. A confocal microscope (Leica, Mannheim, Germany) was used to obtain fluorescence images of TFNAs@PLT.

### Encapsulation efficiency (EE%) and release efficiency evaluation

For the EE% evaluation, fluorescently labeled TFNAs were used to construct TFNAs@PLT. The encapsulation efficiency was calculated based on the fluorescence intensity detected in TFNAs@PLT using the following formula.

EE%
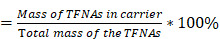


ADP (10 μmol/L) was used as an activator to measure the fluorescence intensity of activated and non-activated TFNAs@PLT, and the release efficiency was calculated accordingly. Fluorescence detection was performed using a Shimadzu RF-5301PC (Japan) spectrofluorophotometer, with fluorescence excitation and emission peaks at 680 nm and 710 nm, respectively.

### Animals

The 8-week-old male C57BL/6 mice (20-22 g) were used for model construction, and the 12-week-old male SPF SD rats (220-250 g) were used for PLT extraction. All experimental animals were purchased from the Experimental Animal Center of Nantong University. The animal studies were approved by the Animal Care and Use Committee of Nanjing Medical University (S20240115-091). The experiments were conducted at Nantong University, and all operations were performed strictly in accordance with ARRIVE2.0 (Animal Research: Reporting of *In Vivo* Experiments 2.0). The animals were kept in an atmosphere of 50-60% humidity and a temperature of 20-25 ºC on a 12 h light:12 h dark cycle. Animals had free access to water.

### Animal model construction and experimental design

The ischemia-reperfusion (I/R) model, typically used in AKI studies, was constructed according to the following protocol. C57BL/6 mice were anesthetized with pentobarbital sodium, placed on a 40 ºC pad, and then injected with 2 mL of warm saline to keep normal body temperature. We injected normal saline (NS) (1 mL) during both the ischemia step and the reperfusion period. The mice were abdominal-midline incised, and both renal arteries were clamped with microvascular clamps (Createmicrobeau Tech Co., Ltd., Shenzhen, China). The clamps were removed after 45 min of blocking, and the incisions were sutured. The operated mice were returned to their cages to recover and wake up with standard feeding. All control mice in each experiment received the same operation without clamping the renal pedicle or ureter ligation.

The model mice were randomly separated into four groups: the blank sham group; the I/R+NS group, in which NS (0.1 mL) was administered after surgery; the I/R+TFNAs group, in which 0.1 mL of the TFNAs was injected; the I/R+TFNAs@PLT group, in which TFNAs@PLT (0.1 mL) were administered. The NS or drug was immediately injected after the incisions were sutured. The blood samples and kidney tissues were collected at specific time points. After separating into upper and lower bilateral parts, the kidneys were immediately transferred to the -80 ºC for storage. A small portion of the middle kidney was collected and fixed in 2.5% glutaraldehyde and 4% paraformaldehyde solutions for subsequent TEM observation and pathological analysis, respectively.

For the chronic nephropathy model, the 8-week-old C57BL/6 mice were injected with adriamycin (ADR) (18 mg/kg) or sterile PBS *via* the tail vein. The occurrence of proteinuria indicated successful construction of the model. Then, the mice were randomly grouped as (1) the sham group; (2) the ADR+NS group; (3) the ADR+TFNAs group, in which 0.1 mL of TFNAs was injected; (4) the ADR+TFNAs@PLT group, in which 0.1 mL of TFNAs@PLT was administered. The treatments were started on the 8^th^ day, and the drugs were given every other day for the next 21 days. The mice were then euthanized, and the tissues were collected three weeks after ADR treatment.

### Detection of urine protein

The successful modeling of AKI and CKD was confirmed by the presence of proteinuria, as indicated by the urine protein-to-creatinine ratio (UPCR) in a spot urine sample. Briefly, Urine was obtained by gently pressing the lower abdomen of mice. Urinary protein concentration was measured using the sulfosalicylic acid method, and urinary creatinine concentration was determined with a commercial kit from Nanjing Jiancheng Company. The UPCR was calculated as follows:

UPCR
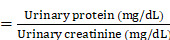


A three-fold or greater increase in UPCR was considered indicative of successful model establishment.

### *In vivo* imaging

To determine whether TFNAs@PLT were delivered into the injured kidney, TFNAs were labeled with Cy5 and used for the synthesis of TFNAs@PLT. Cy5-TFNAs were designed into one chain of the TFNAs as described above, then incubated with PLT to form TFNAs@PLT after sonication. *In vivo* imaging was performed using a PerkinElmer IVIS Lumina Series III *ex/in vivo* imaging system (Waltham, MA, USA). Fluorescence images were obtained after administering TFNAs and TFNAs@PLT for 0.5 h.

### Renal function assay

Serum creatinine (SCr) and blood urea nitrogen (BUN), two key indicators of renal function, were used for evaluation. BUN and SCr levels were measured using commercially available assay kits. ELISA kits were obtained from Nanjing Jiancheng Bioengineering Institute Co., Ltd. Serum was collected and mixed with the kit solutions according to the manufacturer's instructions and incubated for an appropriate time. The supernatant absorbances were measured at 640 nm for BUN levels and 546 nm for SCr levels using the BioTek ELx808 microplate reader system (ThermoFisher Scientific, VT, USA). BUN levels were then obtained according to the manufacturer's instructions. Urea protein levels were also measured by the ELISA kit.

### Cell culture

The human kidney 2 (HK-2) cell line, purchased from ATCC (CRL-2190 ™), was used for *in vitro* TFNAs and TFNAs@PLT function verifications. Cell culture was performed in DMEM supplemented with 10% FBS at 37 ºC, 5% CO_2_. The culture medium was changed every other day. All experiments were conducted when cells reached 80% confluence.

### Lipopolysaccharide (LPS)-induced ROS accumulation and treatment

LPS was added to the human kidney (HK)-2 cells to establish an *in vitro* model of ROS accumulation. Briefly, LPS (1 μg/mL) was added to the HK-2 cells and incubated for 6 h to trigger ROS generation as described in a previous study 18. TFNAs (20 mg/mL) were applied for the TFNAs@PLT construction. According to the encapsulation efficiency, the concentration of TFNAs was nearly 0.72 mg/mL in the final TFNAs@PLT. Then, 3.6 μL TFNAs and 0.1 mL TFNAs@PLT were added to the cells to quench ROS. The cellular ROS levels were stained with dichlorodihydrofluorescein diacetate (DCFH-DA) and measured using a flow cytometer (FCM) according to the kit instructions (S0035S, Beyotime Biotechnology) after treatment.

### Western blotting

The collected PLT and synthesized TFNAs@PLT were lysed with ultrasound (30% power output, pulse cycle: 2 s on and 5 s off for a total of 35 s) on ice, and the proteins were prepared. Subsequently, protein samples (10 μL) were separated on 10% SDS-PAGE gels, followed by electrophoretic transfer to a methanol-activated PVDF membrane. Then the membrane was blocked with non-fat milk and incubated at 4 °C with an anti-CD62P antibody (ab59738, Abcam) overnight. At last, the secondary goat-anti-rabbit antibodies (A0208, Beyotime Biotechnology) were added and incubated for another 1 h. β-actin (AF5003, Beyotime Biotechnology) was used as a loading control.

### Mitochondrial membrane potential assay

HK-2 cells were cultured and treated with H₂O₂ for 6 h, and subsequently incubated with either TFNAs or TFNAs@PLT for 6 h. Following treatment, cells were stained with the JC-1 assay kit per the manufacturer's protocol. Briefly, they were incubated with 5 μg/mL JC-1 dye at 37 °C for 20 min in the dark, followed by three PBS washes prior to image acquisition using a laser scanning confocal microscope. JC-1 monomers were excited at 514 nm, and their emission was detected at 529 nm. JC-1 polymers were excited at 585 nm, and emission was detected at 590 nm.

### ELISA

The tissue IL-6 and TNF-α levels in the mouse kidneys were examined using commercial ELISA kits purchased from Sangon Biotech (D721150-0048 and D721022-0048, respectively). The experiments were conducted per the manufacturer's protocol. Briefly, the kidney tissues were cut into pieces and homogenized using ultrasound (power output: 35%, pulse cycle: 4 s on and 6 s off, repeated 20 times). Then, the supernatant was collected for analysis after the sample was centrifuged. The sample absorbances were obtained at 450 nm, and the levels of IL-6 and TNF-α were obtained using the standard curve.

### TEM

The freshly collected kidney tissues from the mouse models in different treatment groups were blocked and fixed with 2.5% glutaraldehyde (0.1 M phosphate buffer, pH 7.4) at 4 °C for a minimum of 24 h for TEM analysis. 1% OsO_4_ was used for post-fixation at room temperature for 2 h, protected from light. Then, a gradient of diluted ethanol (30%-100%) was used for dehydration, with each step lasting 15-20 min. Subsequently, the tissues were embedded in paraffin, cut into ultra-thin slices, and incubated with uranyl acetate (2%) for 15 min followed by lead citrate (2.6%) for 5 min at room temperature. The morphology of the basal membrane podocytes was observed with the TEM (JEM-200CX, JEOL Co., Japan).

### Immunofluorescence and immunohistochemistry

Following collection, tissues were immediately fixed in 4% paraformaldehyde (in 0.1 M phosphate buffer, pH 7.4) at 4 °C for 24 h. After fixation, they were processed through standard dehydration, clearing, and paraffin embedding, followed by sectioning into ultra-thin slices. Subsequent to deparaffinization and rehydration, antigen retrieval was conducted in citrate buffer (pH 6.0) at 95-100 °C for 15 min. The sections were then blocked with 10% normal goat serum at room temperature for 1 h to minimize nonspecific binding. Immunostaining was performed by incubating with primary antibodies against NLRP3 (ab270449), caspase-1 (ab207802), caspase-3 (ab32351), cleaved-caspase-3 (ab32042), IL-1β (ab315084), GSDME (ab215191), TNF-α (ab183218), and NF-κB/P65 (ab207297) overnight at 4 °C. After PBS washes, the sections were incubated with appropriate secondary antibodies (goat anti-rabbit or rabbit anti-mouse) for 1 h. Finally, images were acquired using an Olympus IX71 light microscope.

### TUNEL assay

TUNEL staining was performed to assess the apoptosis levels in the kidney tissues. Briefly, the kidney slices were incubated in a humid atmosphere at 37 ºC for 1 h. Subsequently, we added the streptavidin-HRP reaction solution, followed by the addition of diaminobenzidine (DAB). Counterstaining was accomplished using hematoxylin. G1507 was applied to label the apoptotic cells with brown to quantify the positive ratio in the TUNEL assay. For quantitative evaluation, 5 independent fields were randomly chosen, and apoptotic cells in glomeruli and renal tubules were recorded. Cells with nuclei exhibiting brown staining, where the staining intensity was significantly higher than the background and nonspecific staining, were identified as TUNEL-positive cells. Only cells adjacent to two bordering sides were included in the count.

### Kidney histology examination and injury assessment

Kidney tissues were fixed in paraformaldehyde, washed with PBS, and embedded in paraffin for ultra-thin sectioning. Pathological changes were evaluated by conventional hematoxylin and eosin (H&E) and periodic acid-Schiff (PAS) staining. Meanwhile, renal tubulointerstitial fibrosis in the CKD mouse model was assessed using Masson and Sirius Red staining, standard methods for collagen fiber detection, performed in accordance with established protocols.

### Renal tubular injury score (TIS)

Tubular injury was scored based on the percentage of cortical and outer medullary tubules exhibiting epithelial necrosis, luminal necrotic debris, or tubular dilatation, according to the following criteria: none = 0; <5% = 1; 5%-30% = 2; 31%-75% = 3; and >75% = 4. For each slide, 10 fields were observed at a magnification of ×200. Histopathological scoring was performed in blinded conditions.

### Determination of blood biochemistry

Liver function biomarkers, including aspartate aminotransferase (AST), alanine aminotransferase (ALT), and globulin (GLB), were measured using the institutional automated biochemical analyzer. The indicators were calculated from the blood samples collected on 1^st^, 7^th^, 14^th^, and 28^th^ days after the administration of TFNAs@PLT and TFNAs.

### Statistical analysis

We used SPSS 20.0 software for the statistical analysis of the results. Data are presented as mean ± SE. Group differences were analyzed by one-way ANOVA, with a P-value of less than 0.05 considered statistically significant.

## Results and Discussion

### Synthesis and characterization of TFNAs@PLT

**Figure 2:**
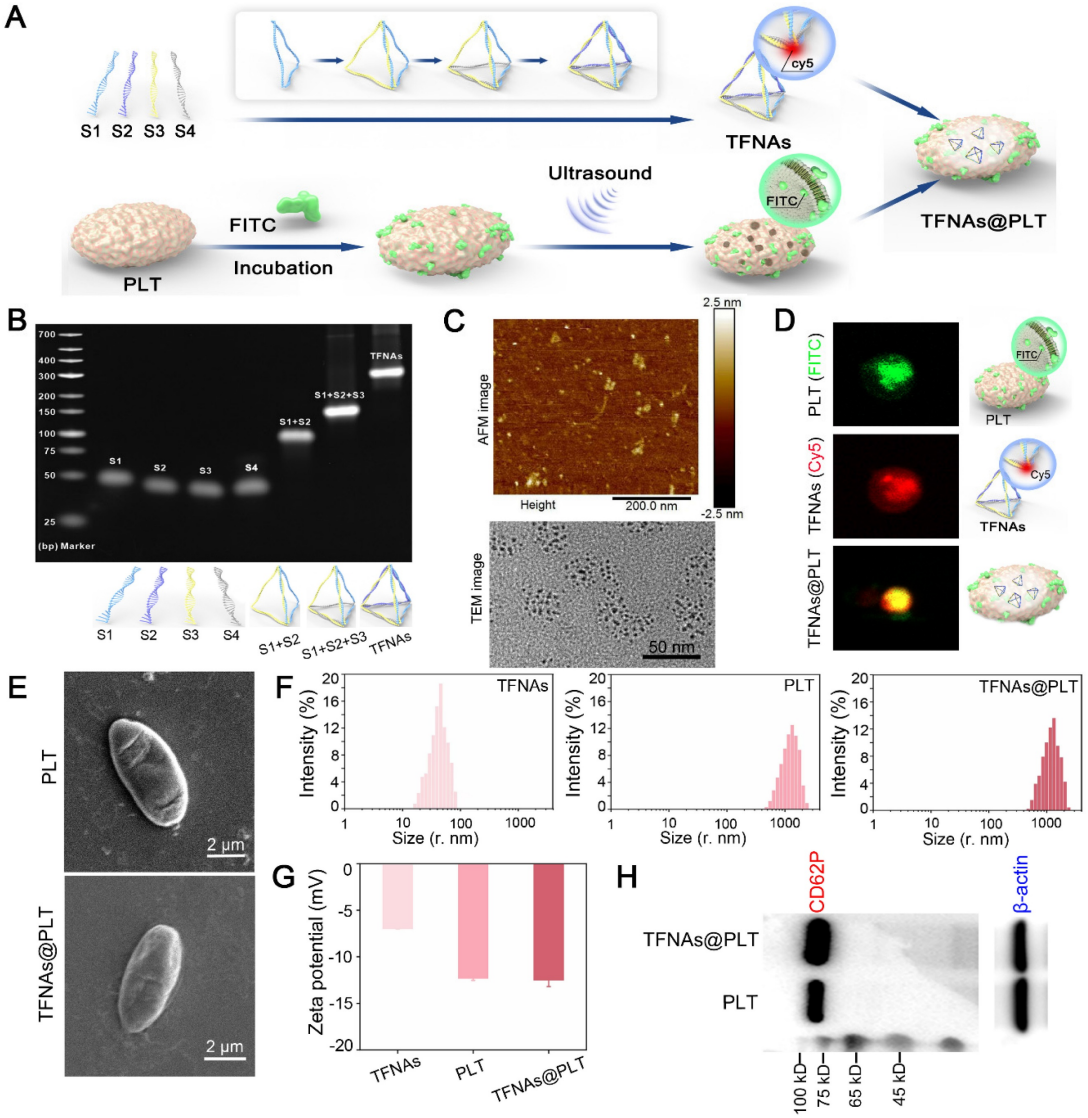
** Synthesis and Characterization of TFNAs@PLT.** (A) Synthesis of TFNAs@PLT (B) DNA agarose gel electrophoresis showed the monomer and intermediates during TFNAs@PLT synthesis. (C) AFM and TEM image of TFNAs@PLT. (D) Fluorescence images of PLT (green) and TFNAs (red), and the TFNAs@PLT (yellow). (E) SEM images of TFNAs and TFNAs@PLT. (F) Size distribution of TFNAs, PLT, TFNAs@PLT. (G) Zeta potential of TFNAs, PLT, TFNAs@PLT. (H) Western blotting results of the specific platelet surface marker (CD62P) in PLT and TFNAs@PLT. β-actin was used as a loading control.

TFNAs were self-constructed with four synthesized DNA strands after one hour of thermal annealing, as illustrated in Figure 2A. DNA agarose gel electrophoresis was utilized to monitor the production. The results (Figure 2B) showed four single strands (S1, S2, S3, and S4), as well as two in-process products and the final TFNAs. Transmission electron microscope (TEM) and atomic force microscope (AFM) images (Figure 2C) revealed that the TFNAs were pyramid-shaped, with a primary size of approximately 5 nm, indicating successful synthesis. As artificially synthesized biomacromolecules, TFNAs exhibit good biocompatibility, strong reducibility, and effective ROS quenching 19. Although TFNAs are relatively stable, they are easily degraded by nucleases or attacked by the immune system 20, and they also lack spatial selectivity, which contributes to their ineffectiveness. Therefore, there is an urgent need to search for suitable carriers to protect them and realize spatial selective delivery 21. Platelet-cloaked carriers have distinct advantages as they can protect TFNAs from degradation and immune system attack *via* the CD47 receptor 22, prolonging their circulation time. They can target injured tissues through their specific surface ligands, enabling specific targeting to injured vascular endothelial cells 23.

For encapsulation, the PLTs were sonicated at 80 W for 10 min to generate tiny pores that permit the entrance of TFNAs 24. TFNAs were then added to PLTs and incubated, allowing their entry into PLTs before the recovery of membrane pores. After that, the samples were transferred to a hyperfiltration tube and centrifuged at 1000 × g for 10 min to separate the unpackaged TFNAs. We performed a co-localization analysis to confirm that TFNA was successfully embedded into PLT. TFNA was labeled red with Cy5, as described above, and the PLTs were stained green with FITC to indicate the presence of TFNAs@PLT. Figure 2D indicates that TFNAs and PLTs presented simultaneous signals with TFNAs@PLT.

We further investigated whether PLTs retained their morphology and functions by examining the performance, size distribution, surface charge, and functional markers. The results indicated that after loading TFNAs, PLTs kept their original morphology (Figure 2E), diameter distribution (Figure 2F), and surface zeta potential (Figure 2G) compared with raw PLTs. CD62P is a crucial cell adhesion molecule on the surface of PLTs that guides the recognition of PLTs to endothelial cell injury 25. Western blotting showed the presence of CD62P in TFNAs@PLT (Figure 2H), indicating that the surface marker and its associated functions were intact. The results indicated that the biological behaviors of PLTs were not affected and could respond to inflammatory signals, allowing them to undergo spatially selective aggregation 26.

### Activation, release, and targeted delivery of TFNAs@PLT

**Figure 3:**
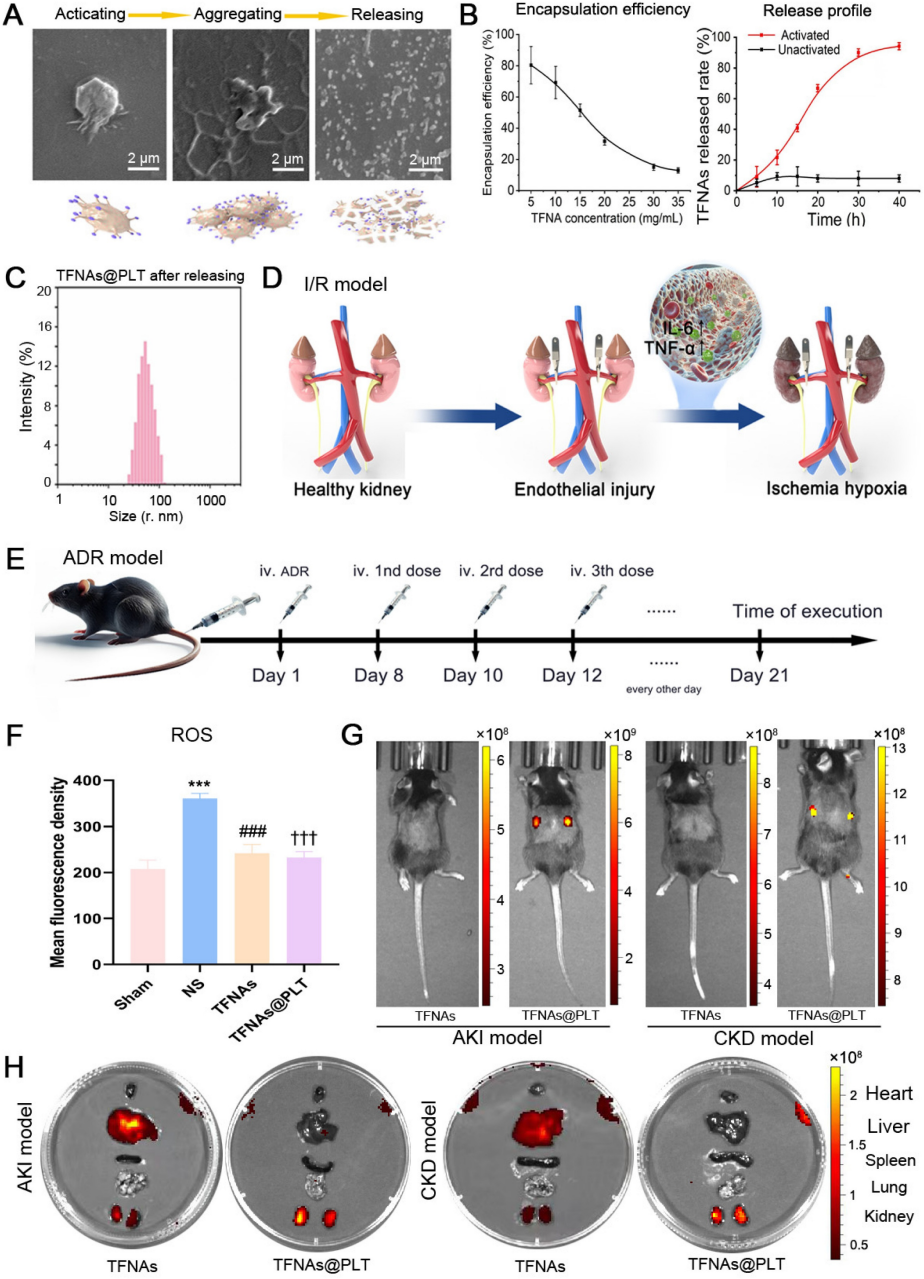
** Activation and monitoring of TFNAs@PLT** (A) SEM images of TFNAs@PLT after TNF-α activation in different stages: magnification × 10,000. (B) Encapsulation efficiency of TFNAs under various concentrations and the TFNAs release profile with time. (C) Size distribution of TFNAs@PLT after activation by TNF-α. (D, E) Acute kidney injury (I/R model) and chronic kidney disease (ADR) mouse models. (F) ROS levels in HK-2 cells examined by FCM after receiving different treatments, *** Sham group vs. NS group, P < 0.001; ^###^ NS group vs. TFNAs group, P < 0.001; **^†††^** NS group vs. TFNAs@PLT group, P < 0.001. The assay was repeated three times. (G, H) Fluorescence images of AKI and CKD mice (n = 3) by the *in vivo* imaging technology after TFNAs@PLT administration at 0.5 h and the accumulation of TFNAs@PLT in the main organs (heart, liver, spleen, lung, and kidney).

The morphological changes of TFNAs@PLT were observed with SEM during activation, following the addition of TNF-α (20 ng/mL) 27, a cytokine that activates PLT 28. As displayed in Figure 3A, TFNAs@PLT initially presented an irregular spherical shape, then developed several tentacles and grew, becoming crossed and connected to form an aggregate. At the final stage, TFNAs@PLT disintegrated into smaller fragments, releasing their contents and facilitating the penetration of TFNAs into injured renal cells. The encapsulation efficiency analysis (Figure 3B) showed that, with an elevated TFNA concentration, the amount of TFNAs entering PLTs decreased, contributing to the reduced encapsulation efficiency. The release profile indicated that the unactivated TFNAs@PLT could stably exist for at least 40 h, and TFNAs were consistently released for approximately 40 h after activation. The dynamic light scattering (DLS) results (Figure 3C) showed that the diameter of the sample changed after TNF-α activation, as indicated by the distribution of TFNAs, confirming successful release.

Figures 3D and 3E illustrate the procedure for creating AKI and CKD mouse models *via* ischemia-reperfusion and AD injection. To further demonstrate the ROS elimination ability of TFNAs@PLT, a LPS-induced ROS burst cell model was employed. The FCM results (Figure 3F) showed that the ROS level was significantly decreased (P < 0.001) after the application of TFNAs and TFNAs@PLT, suggesting that both possess strong antioxidant abilities *in vitro*. Excessive generation and dysregulation of ROS are known to be critical characteristics of both AKI and CKD 29, and studies have proven that inhibiting ROS can effectively mitigate the damage caused by AKI 30, 31. To verify the targeted delivery of TFNAs@PLT to the injured kidney *in vivo*, the biodistribution profiles of TFNAs@PLT were observed using Cy5 signals after injecting them into the model mice. Cy5 signals exhibited non-targeted accumulation in control mice, while they were significantly increased in the kidney region of the AKI and CKD mouse models (Figure 3G). Furthermore, the images (Figure 3H) of the main organs (heart, liver, lung, spleen, and kidney) showed that TFNAs mainly accumulated in the blood-rich liver, with lesser amounts in the kidneys. In contrast, the TFNAs@PLT were primarily accumulated in the injured kidney after redistribution, providing evidence of the spatially selective accumulation of TFNAs@PLT in the injured kidneys for therapeutic purposes.

### Therapeutic effects of TFNAs@PLT on acute kidney injury *in vivo*

**Figure 4:**
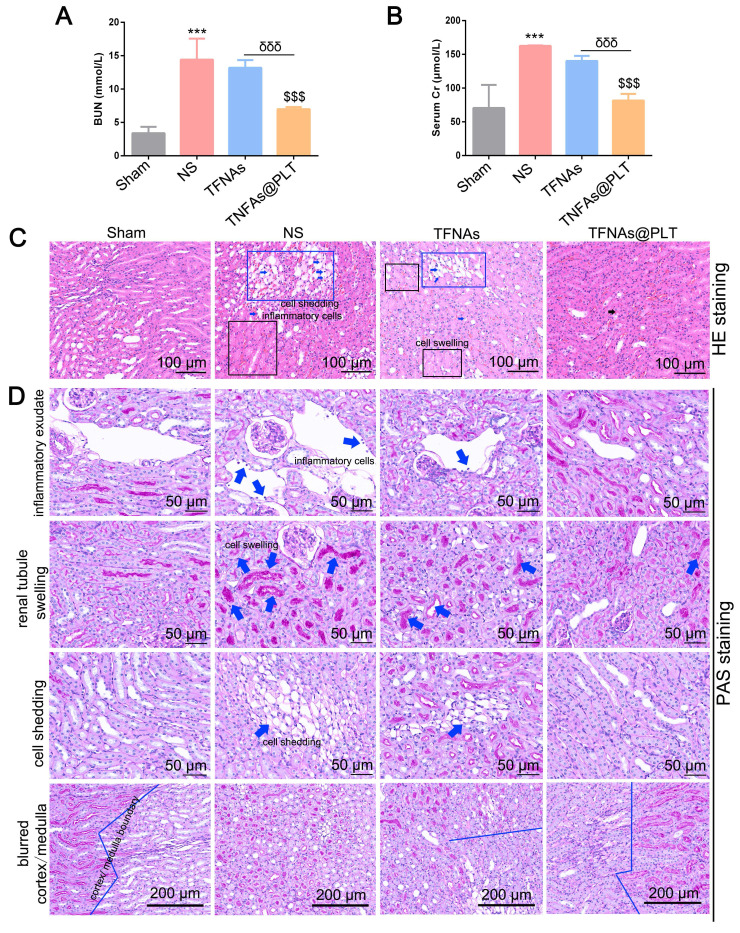
** TFNAs@PLT against AKI.** (A, B) Concentrations of urea nitrogen (BUN) and serum creatinine (SCr) were measured after mice (n = 3) received various treatments for 24 h in three independent experiments. *** Sham group vs. NS group, *P <* 0.001; $$$ NS group vs. TFNAs@PLT group, *P <* 0.001; δδδ TFNAs vs. TFNAs@PLT group, *P <* 0.001. (C, D) The H&E and PAS staining of kidney tissues collected from mice (n = 3) were examined after modeling and treatment with TFNAs and TFNAs@PLT. Pathological changes are indicated with arrows or squares along with annotations.

To evaluate the therapeutic effect of TFNAs@PLT on AKI, the concentrations of BUN and SCr, key indicators of kidney function, were first examined. The results (Figure 4A-B) showed that BUN and SCr levels were significantly increased (*P <* 0.001) after modeling, and the administration of TFNAs@PLT significantly reduced BUN and SCr levels (*P <* 0.001). The exfoliated nucleus, cell swelling, and cell shedding were observed in the renal tubular lumen by H&E staining (Figure 4C), as previously reported in the I/R model 32. The pathological changes were mostly rescued after TFNAs@PLT treatment. The subsequent tubular injury scoring (TIS) evaluation showed consistent results with the pathology changes (Figure S1). Further, PAS staining (Figure 4D) indicated that inflammatory exudate, cell swelling, cell shedding, and the blurred cortex/medulla boundary were substantially rescued after the administration of TFNAs@PLT.

**Figure 5:**
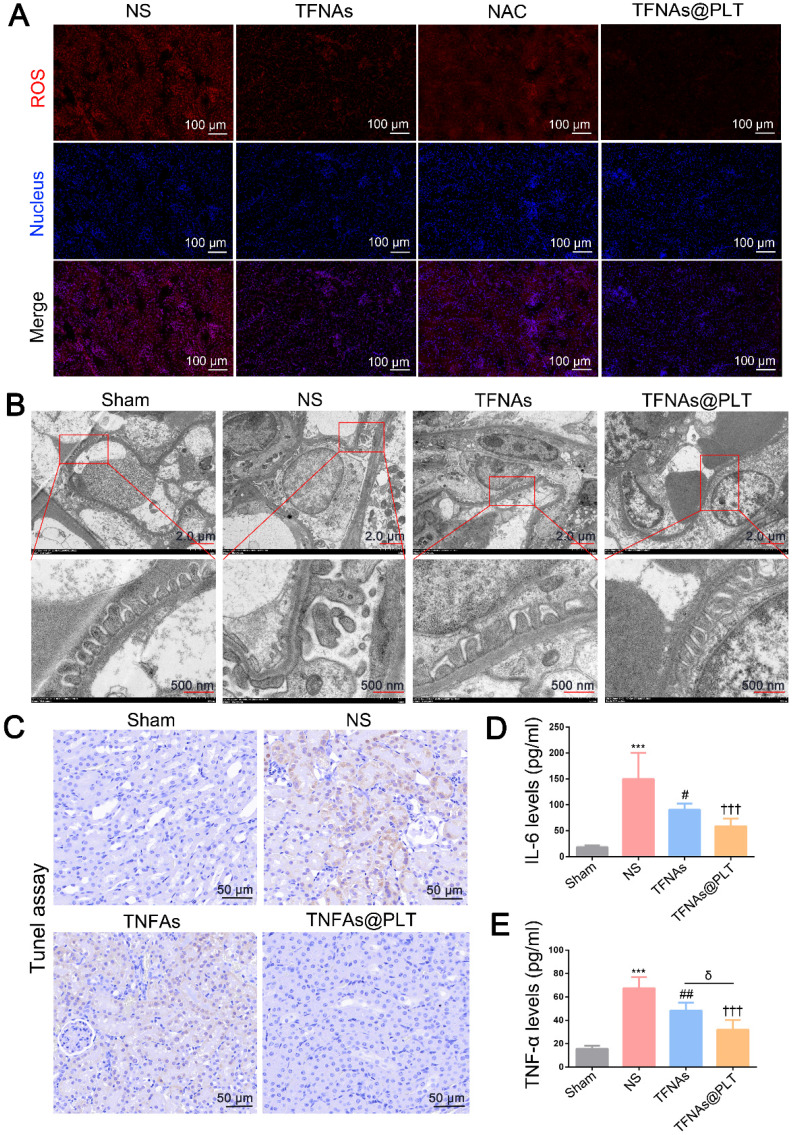
** Therapeutic effects of TFNAs@PLT on the I/R mouse model** (A) ROS levels were examined by immunofluorescence after the model mice received various treatments (n = 3). (B) TEM images of kidney tissues following treatment with NS, TFNAs, and TFNAs@PLT; the specific sites with obvious changes are amplified. (C) Apoptosis levels were evaluated with the TUNEL assay after corresponding treatments. (D, E) IL-6 and TNF-α levels were measured with an ELISA kit after various treatments (n = 3), *** Sham group vs. NS group, *P <* 0.001; ## NS group vs. TFNAs group, *P <* 0.01; **†††** NS group vs. TFNAs@PLT group, *P <* 0.001; δ TFNAs group vs. TFNAs@PLT group, *P <* 0.05. The assay was conducted three times.

Podocyte impairment has been recognized as a vital feature of AKI, and ROS has been proven to be a key factor in podocyte damage 31. Therefore, TFNAs are likely to rescue podocyte injury *via* quenching ROS. We employed the typical ROS clearance agent N-acetylcysteine (NAC) to verify this hypothesis and evaluate the antioxidant ability of TFNAs@PLT. The location of the glomerular basement membrane was also monitored by TEM at 24 h after different treatments. The results (Figure 5A) indicated that the ROS level was significantly increased in the AKI mouse model, and TFNAs presented a better antioxidant effect than NAC, which was less effective than that of TFNAs@PLT based on the spatial delivery. TEM images (Figure 5B) revealed severe podocyte damage in the I/R mouse model, characterized by shortened or diminished foot processes in kidney tissues.

In contrast, treatment with TFNAs@PLT markedly restored the structure of the foot processes. The inflammation-mediated cell apoptosis and pyroptosis were considered key features of AKI following TNF-α activation, with IL-6 serving as a key indicator for mitochondrial damage 33. To determine whether the protective effects of TFNAs@PLT on AKI are related to mitochondrial impairment, we first examined changes in mitochondrial morphology and membrane potential. Subsequently, pyroptosis and inflammation levels were assessed in renal tissues using the TUNEL assay and by analyzing the expression of IL-6 and TNF-α. Figure S2 shows that, although the mitochondrial morphology did not exhibit any apparent changes, the mitochondrial membrane potential was rescued by TFNAs@PLT. As presented in Figure 5C, the ratio of the TUNEL-positive cells was significantly decreased in the TFNAs@PLT group compared with the NS group, and IL-6 and TNF-α levels significantly declined (*P <* 0.001) after TFNAs@PLT treatment (Figure 5D-E).

### TFNAs@PLT relieves the acute kidney injury damage in the I/R mouse model

**Figure 6:**
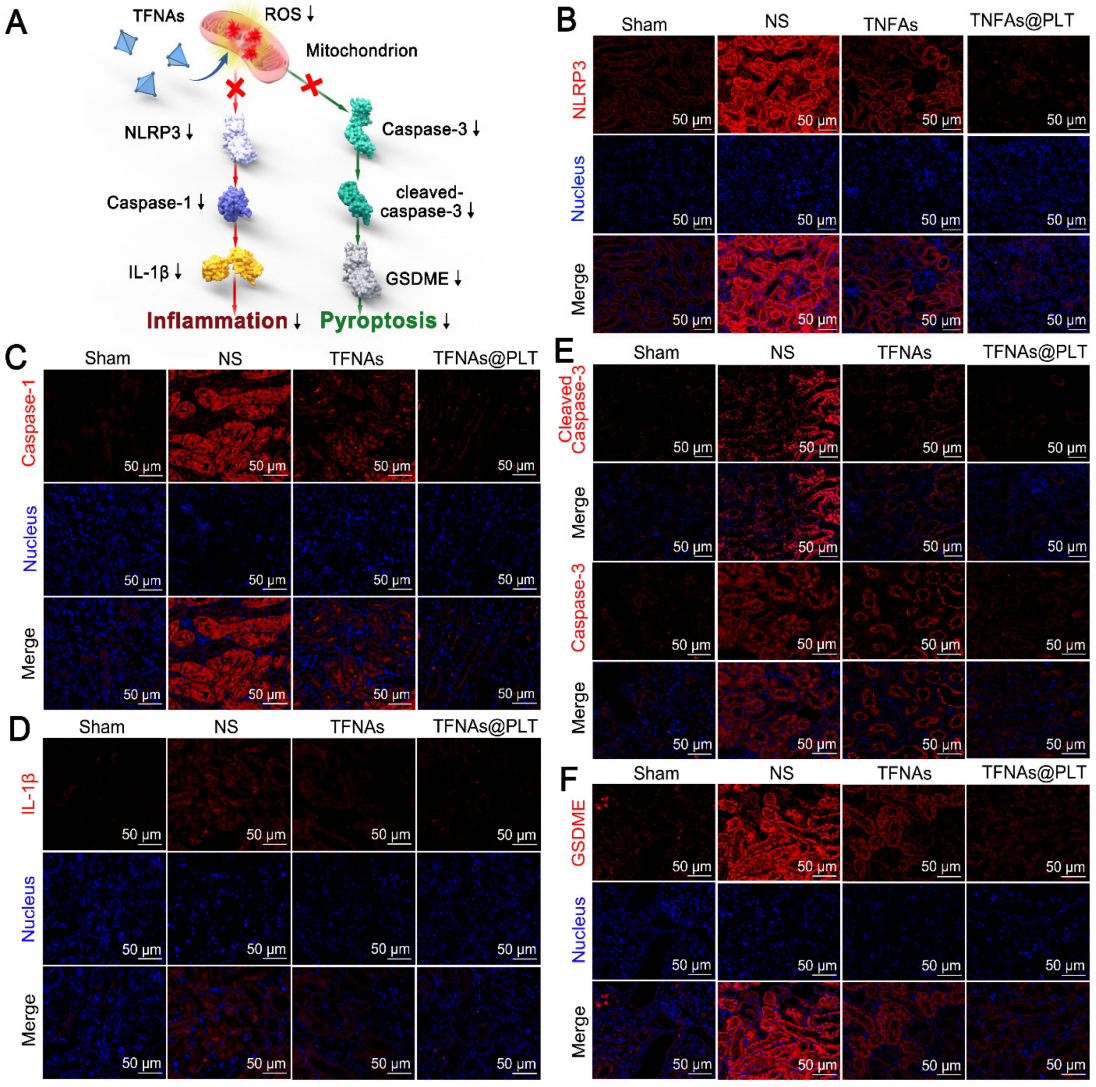
** Therapeutic effects of TFNAs@PLT on AKI.** (A) Schematic illustration showing the pathways TFNAs@PLT use against kidney injury. (B-F) Key protein expression levels of NLRP3, caspase-1, and IL-1β in the NLRP3/Caspase-1/IL-1β pathway, and the expression of caspase-3, cleaved caspase-3, and GSDME in the caspase-3/GSDME pathway were examined by immunofluorescence after modeling and treatment of TFNAs and TFNAs@PLT (n = 3). All target proteins were stained red with CY5-labeled secondary antibody, and the nucleus was stained blue with DAPI.

Ischemia is known for leading to a burst of ROS in kidney cells, which in turn impairs mitochondrial function, thereby activating inflammation-mediated apoptosis and pyroptosis 34. Caspase-3/GSDME and the NLRP3/Caspase-1/IL-1β are typical pathways that regulate pyroptosis and apoptosis, as well as manage the switch of apoptosis to pyroptosis 35, 36. We examined whether TFNAs@PLT could alleviate kidney injury by reducing apoptosis and inhibiting ROS. Figure 6A presents the speculative mechanical framework, and Figures 6B-D show that NLRP3 was activated in the AKI mouse model, leading to subsequent caspase-1 activation and a significant increase in IL-1β levels, further promoting inflammation activation. However, when TFNAs@PLT inhibited ROS, NLRP3 expression was downregulated, resulting in the inhibition of the Caspase-1/IL-1β pathway and partial mitigation of kidney injury.

The NLRP3-mediated Caspase-1/IL-1β pathway is a typical route that regulates inflammation-mediated apoptosis and pyroptosis 37, 38, and can be triggered by ROS. Therefore, the antioxidant ability of TFNAs can effectively inhibit this pathway and relieve kidney injury. Pyroptosis is reported to be involved in many inflammatory diseases, in which GSDME plays a crucial role. Additionally, the deletion of the GSDME gene has been reported to protect the kidney against ischemia-reperfusion-induced injury through the caspase-3/GSDME pathway 39, 40. Therefore, we examined the caspase-3/GSDME pathway and found caspase-3, cleaved caspase 3, and GSDME expression levels to be significantly increased to regulate pyroptosis in the I/R mice (Figure 6E-F). Also, the application of TFNAs@PLT inhibited their activation and protected against kidney injury.

### TFNAs@PLT mitigates renal fibrosis in the ADR mouse model

**Figure 7:**
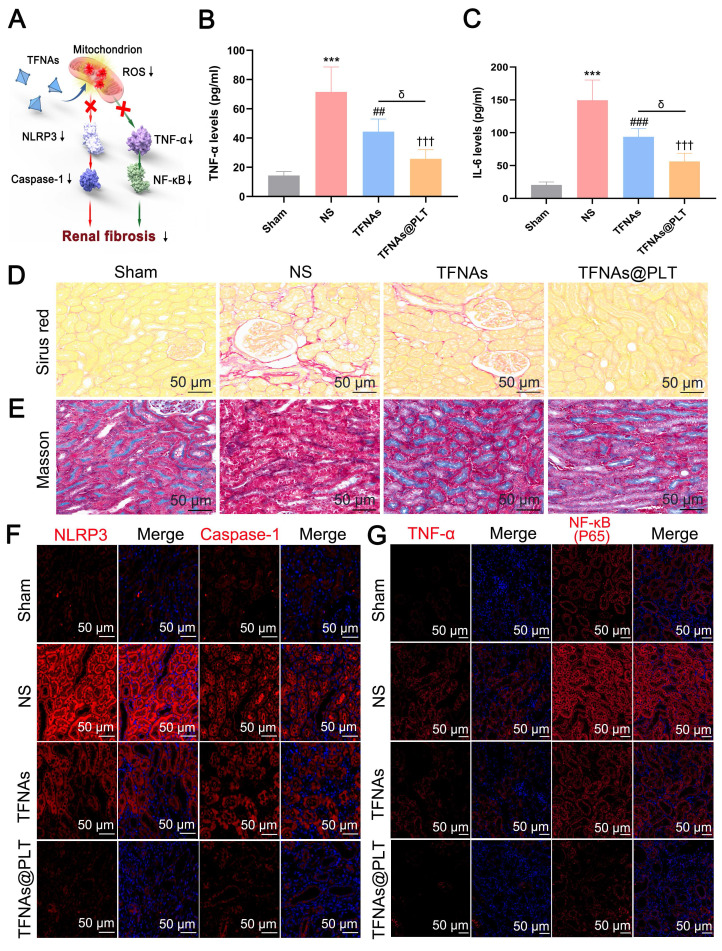
** Therapeutic effects of TFNAs@PLT on CKD** (A) Proposed TFNAs@PLT mechanism against kidney fibrosis in the CKD model. (B & C) IL-6 and TNF-α levels in the CKD mouse model were measured by ELISA after treatment with NS, TFNAs, and TFNAs@PLT. *** Sham group vs. NS group, *P <* 0.001; ## NS group vs. TFNAs group, *P <* 0.01; **†††** NS group vs. TFNAs@PLT group, *P <* 0.001; δ TFNAs group vs. TFNAs@PLT group, *P <* 0.05. The results are from three independent studies. (D & E) Renal fibrosis levels after different treatments were evaluated with Sirius red staining and Masson staining. (F & G) Protein expression levels of NLRP3, caspase-1, TNF-α, and NF-κB were determined by immunofluorescence after the mice (n = 3) received corresponding treatments.

As has been reported, the untimely or improper treatment for AKI may contribute to chronic kidney disease (CKD) 41, with progressive fibrosis as its main feature 42. Therefore, we first examined TNF-α and IL-6 expression levels, which are markers of the inflammatory reaction, and were a precondition for TFNAs@PLT activation in the ADR mouse model. Figure 7A presents the key pathways that we hypothesized regulate kidney fibrosis. The results (Figures 7B-C) showed that TNF-α and IL-6 levels were significantly (*P <* 0.001) increased in the NS group, and their levels were significantly decreased (*P <* 0.001) after TFNAs@PLT treatment. Kidney interstitial fibrosis is characterized by the abnormal deposition of extracellular matrix, typically mediated by myofibroblasts. Since fibronectin and collagen I are considered the primary components of the extracellular matrix, Sirius Red was used to assess fibrosis levels. The results (Figure 7D) indicated that the red area was increased in the ADR mouse model, decreased in the TFNAs group, and further diminished after TFNAs@PLT treatment, indicating that TFNAs@PLT significantly reduced the fibrosis level of the injured kidney. We next employed Masson staining, a typical method to reveal collagen fibers, to evaluate the renal fibrosis level and determine whether TFNAs@PLT had protective effects on kidney fibrosis during CKD in the ADR mouse model. The positive ratio, indicated by the red area, is the degree of fibrosis within the entire visual field. The results (Figure 7E) showed increased red-stained areas in the interstitium of the ADR mouse model, and the fibrosis level was relatively reduced after treatment with TFNAs@PLT.

To further verify the fibrosis level in the CKD model, two key markers, α-smooth muscle actin (α-SMA) and fibronectin (FN), were examined. The results showed that α-SMA and FN levels were decreased after TFNAs@PLT treatment (Figure S3), consistent with Sirus and Masson staining. Furthermore, to elucidate the anti-fibrosis mechanisms of TFNAs@PLT, we investigated the NLRP3/caspase-1 and TNF-α/NF-κB pathways that regulate renal fibrosis. The results (Figures 7F-G) indicated that the expression levels of caspase-1, NLRP3, TNF-α, and NF-κB were decreased after treatment relative to the control group. Moreover, the decrease in the TFNAs@PLT group was more significant than that in the TFNAs group, suggesting that the NLRP3/caspase-1 and TNF-α/NF-κB pathways may play crucial roles in regulating kidney fibrosis.

Thus, we preliminarily explored the therapeutic effects of TFNAs@PLT on AKI and CKD, and the potential molecular mechanisms involved. However, the regulatory effects of these molecules were not rigorously validated in cellular models. This represents a shortcoming of the current study and will be a primary direction for our future research, which is expected to advance the understanding of the mechanisms underlying TFNAs@PLT and accelerate its clinical translation.

### Bio-safety of TFNAs@PLT *in vivo*

**Figure 8:**
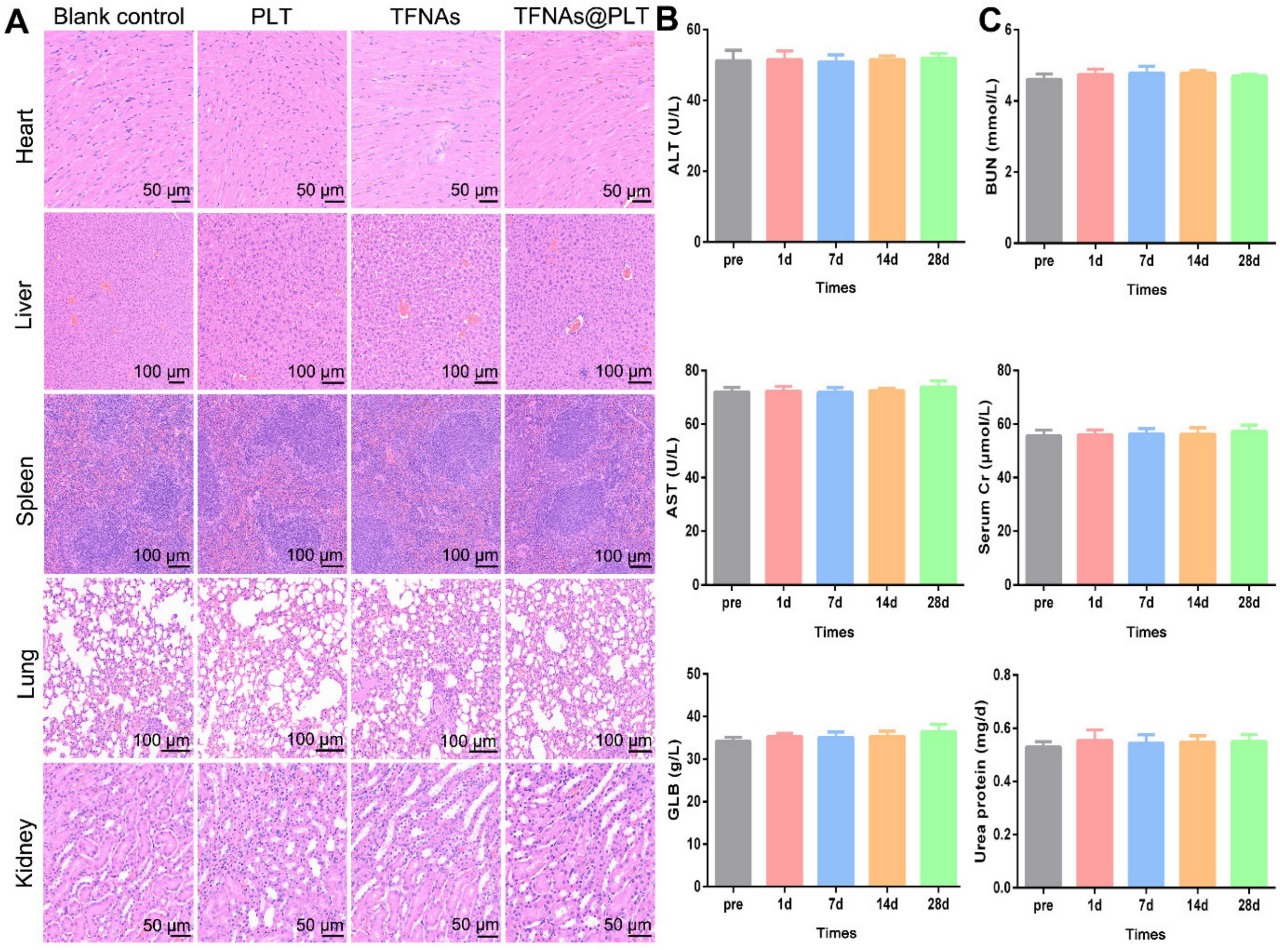
** Safety evaluation of TFNAs@PLT.** (A) Pathology changes in main organs, including the heart, liver, spleen, lungs, and kidneys, were assessed via H&E staining on the 28^th^ day after mice were treated with PLT, TFNAs, or TFNAs@PLT. (B) Indicators reflecting liver function (ALT, AST, and GLB) were evaluated pre-treatment and on the 1^st^, 7^th^, 14^th^, and 28^th^ day after treatment. (C) Key biochemical parameters of renal function, including BUN, SCr, and urinary protein, were also determined at the same time points as liver functions (n = 3). All data are shown as mean ± SD.

Bio-safety is the most crucial aspect for nano-medicine, besides targeted delivery and selective treatment. Therefore, the safety of TFNAs@PLT was assessed in normal mice. Following intravenous administration *via* the tail vein, blood and major organs were harvested on the scheduled days. The results showed no obvious inflammation after TFNAs@PLT treatment in the heart, liver, lung, spleen, and kidney (Figure 8A). Meanwhile, indices indicating liver function (ALT, GLB, and AST) and kidney function (urea protein, SCr, and BUN) were examined. No significant change in the blood biochemistry indicators was observed after TFNAs@PLT treatment from day 0 to day 28 (Figure 8B-C), indicating the good biocompatibility and low toxicity of TFNAs@PLT.

The safety of nanodrugs is a significant concern during the development of nanomedicines. Therefore, we evaluated the safety of TFNAs@PLT in the mouse models. The heart, liver, kidneys, and lungs, which have the richest blood flow, are considered the primary distribution sites in a two-compartment model for drug distribution. Our study found that TFNAs@PLT did not induce visible pathological changes in these organs, suggesting that it delivered the TFNAs to the injured cells effectively and was relatively safe to the healthy cells. In addition to the unchanged pathology, biomarkers for evaluating the liver and renal function were also evaluated, as these organs are crucial for drug metabolism and excretion. Our research revealed no impact on biochemical indicators of liver and kidney function, consistent with previous studies, which further confirmed the safety of PLT.

Though our findings demonstrated the promising potential of the TFNAs@PLT platform for kidney injury therapy, several critical challenges must be addressed before its successful clinical translation. TFNAs@PLT exhibited excellent biocompatibility and intrinsic inflammation-targeting ability. However, its delivery efficiency in complex *in vivo* environments may still need further validation through large animal models. Although the immunogenicity risk of platelet membrane components is relatively low, it is still necessary to ensure that they are derived from allogeneic or autologous sources to minimize the risk of immune reactions. Scalable mass production and quality control pose other challenges for the clinical translation of TFNAs@PLT. Furthermore, efficient methods for obtaining large quantities of consistent platelet membranes and improving coating efficiency are urgent issues that need to be addressed.

## Conclusions

Our study introduces a novel therapeutic strategy for the spatially selective clearance of ROS in injured kidney cells using platelet-cloaked tetrahedral framework nucleic acids (TFNAs@PLT). By leveraging the inherent targeting capabilities of platelets and their responsiveness to inflammatory signals, this system effectively delivers TFNAs to areas of renal injury. We demonstrated that TFNAs@PLT significantly reduce apoptosis and pyroptosis in acute kidney injury models by engaging key inflammatory pathways while also inhibiting renal fibrosis in chronic kidney disease models. Importantly, this approach showcases not only the potential for treating kidney injuries but also offers a blueprint for addressing other inflammation-mediated disorders.

## Supplementary Material

Supplementary figures.

## References

[1] Liu T, Xiao B, Xiang F, Tan J, Chen Z, Zhang X (2020). Small copper-based nanoparticles for reactive oxygen species scavenging and alleviation of inflammation related diseases. Nat Commun.

[2] Racanelli AC, Kikkers SA, Choi AMK, Cloonan SM (2018). Autophagy and inflammation in chronic respiratory disease. Autophagy.

[3] Filomeni G, De Zio D, Cecconi F (2015). Oxidative stress and autophagy: the clash between damage and metabolic needs. Cell Death Differ.

[4] Chen Y, Xu J, Shi S, Ma W, Cui W, Yan R (2024). A DNA nanostructure-Hif-1alpha inducer complex as novel nanotherapy against cisplatin-induced acute kidney injury. Cell Prolif.

[5] Yang Z, Shi L, Wang Y, Zhou D, Zhang C, Lin Y (2024). Unveiling the Potential of Tetrahedral DNA Frameworks in Clinical Medicine: Mechanisms, Advances, and Future Perspectives. Small.

[6] Li X, Li L, Fu X, Huang S, Wang Y, Yang Y (2025). A novel tetrahedral framework nucleic acid-derived chemodynamic therapy agent for effective glioblastoma treatment. Cell Prolif.

[7] Zhang T, Tian T, Lin Y (2022). Functionalizing Framework Nucleic-Acid-Based Nanostructures for Biomedical Application. Adv Mater.

[8] Lan Y, Li X, Liu B, Lu J, Zuo B, Wang Y (2024). Framework nucleic acid-based nanoparticles enhance temozolomide sensitivity in glioblastoma. Drug Resist Updat.

[9] Li X, Lan Y, Fu X, Luo X, Chen J, Zhang W (2024). DNA nanomachine-driven chemodynamic therapy against glioblastoma. Aggregate.

[10] Rezaei B, Harun A, Wu X, Iyer PR, Mostufa S, Ciannella S (2024). Effect of Polymer and Cell Membrane Coatings on Theranostic Applications of Nanoparticles: A Review. Adv Healthc Mater.

[11] Jiang Q, Wang K, Zhang X, Ouyang B, Liu H, Pang Z (2020). Platelet Membrane-Camouflaged Magnetic Nanoparticles for Ferroptosis-Enhanced Cancer Immunotherapy. Small.

[12] Xu X, Li M, Yu F, Wei Q, Liu Y, Tong J (2024). Platelet Membrane Nanocarriers Cascade Targeting Delivery System to Improve Myocardial Remodeling Post Myocardial Ischemia-Reperfusion Injury. Adv Sci (Weinh).

[13] Vennekens A, Laporte E, Hermans F, Cox B, Modave E, Janiszewski A (2021). Kobayashi H, Malengier-Devlies B, Chappell J, et al: Interleukin-6 is an activator of pituitary stem cells upon local damage, a competence quenched in the aging gland. Proc Natl Acad Sci U S A.

[14] Chadwick W, Magnus T, Martin B, Keselman A, Mattson MP, Maudsley S (2008). Targeting TNF-alpha receptors for neurotherapeutics. Trends Neurosci.

[15] Fu L, Li P, Zhu J, Liao Z, Gao C, Li H (2022). Tetrahedral framework nucleic acids promote the biological functions and related mechanism of synovium-derived mesenchymal stem cells and show improved articular cartilage regeneration activity *in situ*. Bioact Mater.

[16] Ding Y, Ge M, Zhang C, Yu J, Xia D, He J (2023). Platelets as delivery vehicles for targeted enrichment of NO(.) to cerebral glioma for magnetic resonance imaging. J Nanobiotechnology.

[17] Tian J, Gao M, Zhu J, Xu H, Ji H, Xia D (2024). Platelets camouflaged nanovehicle improved bladder cancer immunotherapy by triggering pyroptosis. Theranostics.

[18] Wang Y, Lv W, Ma X, Diao R, Luo X, Shen Q (2024). NDUFS3 alleviates oxidative stress and ferroptosis in sepsis induced acute kidney injury through AMPK pathway. Int Immunopharmacol.

[19] Yao Y, Lei X, Wang Y, Zhang G, Huang H, Zhao Y (2023). A Mitochondrial Nanoguard Modulates Redox Homeostasis and Bioenergy Metabolism in Diabetic Peripheral Neuropathy. ACS Nano.

[20] Martin DA, Elkon KB (2006). Intracellular mammalian DNA stimulates myeloid dendritic cells to produce type I interferons predominantly through a toll-like receptor 9-independent pathway. Arthritis Rheum.

[21] Yan Y, Liu XY, Lu A, Wang XY, Jiang LX, Wang JC (2022). Non-viral vectors for RNA delivery. J Control Release.

[22] Li M, Li J, Chen J, Liu Y, Cheng X, Yang F (2020). Platelet Membrane Biomimetic Magnetic Nanocarriers for Targeted Delivery and *in Situ* Generation of Nitric Oxide in Early Ischemic Stroke. ACS Nano.

[23] Zhou T, Yang X, Wang T, Xu M, Huang Z, Yu R (2022). Platelet-Membrane-Encapsulated Carvedilol with Improved Targeting Ability for Relieving Myocardial Ischemia-Reperfusion Injury. Membranes (Basel).

[24] Tharkar P, Varanasi R, Wong WSF, Jin CT, Chrzanowski W (2019). Nano-Enhanced Drug Delivery and Therapeutic Ultrasound for Cancer Treatment and Beyond. Front Bioeng Biotechnol.

[25] Ding H, Cao XY, Ma XG, Zhou WJ (2013). Endothelial cell injury with inflammatory cytokine and coagulation in patients with sepsis. World J Emerg Med.

[26] Gong H, Zhang L, Ma Y, Gui Y, Xiang T, Liu J (2023). Platelet shipped IL-10 enhances drug delivery for attenuating I/R-or UUO-induced renal injury. Chem Eng J.

[27] Zhang L, Gong H, Gong X, Zhou B, Wang X, Fei S (2025). Bioengineered platelet nanoplatform enables renal-targeted dexamethasone delivery for chronic nephritis therapy with dual anti-inflammatory/anti-fibrotic effects and minimized systemic toxicity. Bioact Mater.

[28] Rutella S, Vetrano S, Correale C, Graziani C, Sturm A, Spinelli A (2011). Enhanced platelet adhesion induces angiogenesis in intestinal inflammation and inflammatory bowel disease microvasculature. J Cell Mol Med.

[29] Su L, Zhang J, Gomez H, Kellum JA, Peng Z (2023). Mitochondria ROS and mitophagy in acute kidney injury. Autophagy.

[30] Wu J, Shang H, Zhang A, He Y, Tong Y, Huang Q (2023). Antioxidant nanozymes in kidney injury: mechanism and application. Nanoscale.

[31] Yu H, Jin F, Liu D, Shu G, Wang X, Qi J (2020). ROS-responsive nano-drug delivery system combining mitochondria-targeting ceria nanoparticles with atorvastatin for acute kidney injury. Theranostics.

[32] Basile DP, Anderson MD, Sutton TA (2012). Pathophysiology of acute kidney injury. Compr Physiol.

[33] Jia ZC, Liu SJ, Chen TF, Shi ZZ, Li XL, Gao ZW (2024). Chlorogenic acid can improve spermatogenic dysfunction in rats with varicocele by regulating mitochondrial homeostasis and inhibiting the activation of NLRP3 inflammasomes by oxidative mitochondrial DNA and cGAS/STING pathway. Bioorg Chem.

[34] Fu Y, Cao J, Wei X, Ge Y, Su Z, Yu D (2023). Klotho alleviates contrast-induced acute kidney injury by suppressing oxidative stress, inflammation, and NF-KappaB/NLRP3-mediated pyroptosis. Int Immunopharmacol.

[35] Xiao C, Zhao H, Zhu H, Zhang Y, Su Q, Zhao F (2020). Tisp40 Induces Tubular Epithelial Cell GSDMD-Mediated Pyroptosis in Renal Ischemia-Reperfusion Injury via NF-kappaB Signaling. Front Physiol.

[36] Jiang M, Qi L, Li L, Li Y (2020). The caspase-3/GSDME signal pathway as a switch between apoptosis and pyroptosis in cancer. Cell Death Discov.

[37] Fouad AA, Abdel-Aziz AM, Hamouda AAH (2020). Diacerein Downregulates NLRP3/Caspase-1/IL-1beta and IL-6/STAT3 Pathways of Inflammation and Apoptosis in a Rat Model of Cadmium Testicular Toxicity. Biol Trace Elem Res.

[38] Hang Y, Tan L, Chen Q, Liu Q, Jin Y (2021). E3 ubiquitin ligase TRIM24 deficiency promotes NLRP3/caspase-1/IL-1beta-mediated pyroptosis in endometriosis. Cell Biol Int.

[39] Wang Y, Gao W, Shi X, Ding J, Liu W, He H (2017). Chemotherapy drugs induce pyroptosis through caspase-3 cleavage of a gasdermin. Nature.

[40] Challis P, Nydert P, Hakansson S, Norman M (2021). Association of Adherence to Surfactant Best Practice Uses With Clinical Outcomes Among Neonates in Sweden. JAMA Netw Open.

[41] Wang Z, Zhang C (2022). From AKI to CKD: Maladaptive Repair and the Underlying Mechanisms. Int J Mol Sci.

[42] Huang R, Fu P, Ma L (2023). Kidney fibrosis: from mechanisms to therapeutic medicines. Signal Transduct Target Ther.

