# Supplementary material for: Spatially selective clearance of inflammation-mediated reactive oxygen species in injured kidney cells
Source: Theranostics. 2026 Jan 1;16(3):1466–81. doi: 10.7150/thno.112905 (PMC12679581; doi:10.7150/thno.112905)
Supplement: Supplementary file 1 — Supplementary figures. [file thnov16p1466s1.pdf]

Supplementary Materials

Table S1. Sequences of four DNA strands used for construction of TFNAs

Strands	Sequences (5'-3')
S1	ATTTATCACCCGCCATAGTAGACGTATCACCAGGCAGTTGAGACGAACATTCCTAAGTCTGAA
S2	ACATGCGAGGGTCCAATACCGACGATTACAGCTTGCTACACGATTCAGACTTAGGAATGTTCG
S3	ACTACTATGGCGGGTGATAAAACGTGTAGCAAGCTGTAATCGACGGGAAGAGCATGCCCATCC
S4	ACGGTATTGGACCCTCGCATGACTCAACTGCCTGGTGATACGAGGATGGGCATGCTCTTCCCG

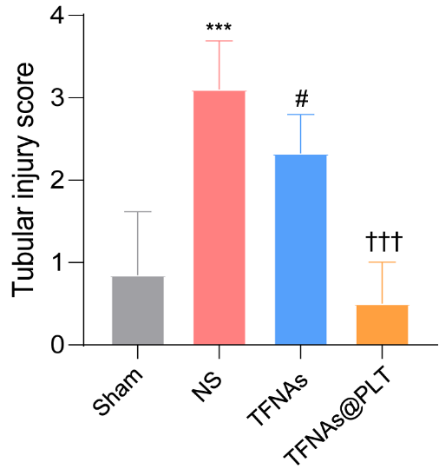

Figure S1. Tubular injury scoring (TIS) was carried out to quantify the impairment and therapeutic effects of TFNAs and TFNAs@PLT on kidney injury. The injuries were observed from 10 independent fields, and the data were presented as mean ± SD, *** Sham group vs. NS group, $P < 0.001$; # NS group vs. TFNAs group, $P < 0.05$; ††† NS group vs. TFNAs@PLT group, $P < 0.001$.

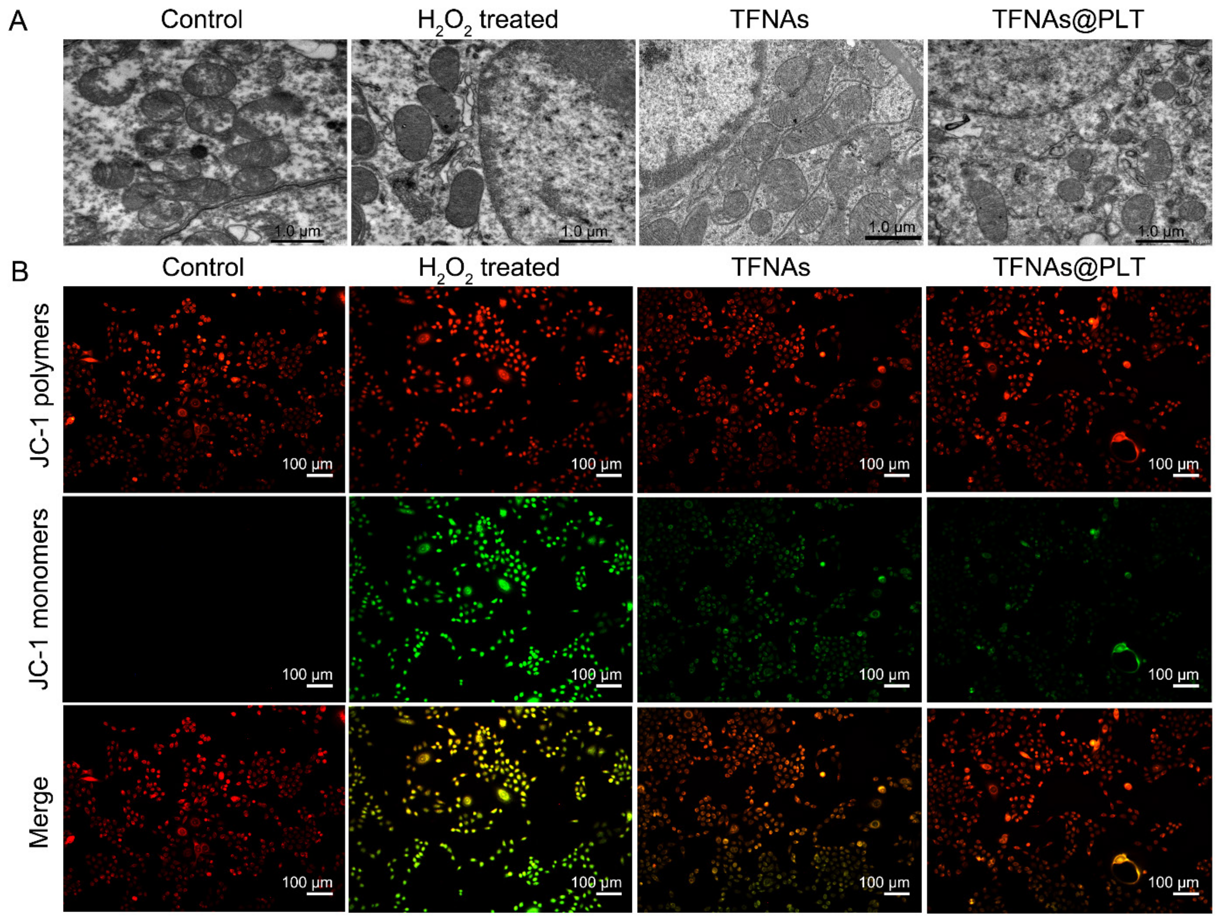

Figure S2: Effects of TFNAs@PLT on H_2O_2-induced ROS in the mitochondria (A) Morphology of the mitochondria was observed by TEM after HK-2 cells were treated with H_2O_2, TFNAs, and TFNAs@PLT. (B) Mitochondrial membrane potentials were revealed by JC-1 after the HK-2 cells were treated with H_2O_2, TFNAs, and TFNAs@PLT. JC-1 polymers and monomers are denoted as red and green, respectively.

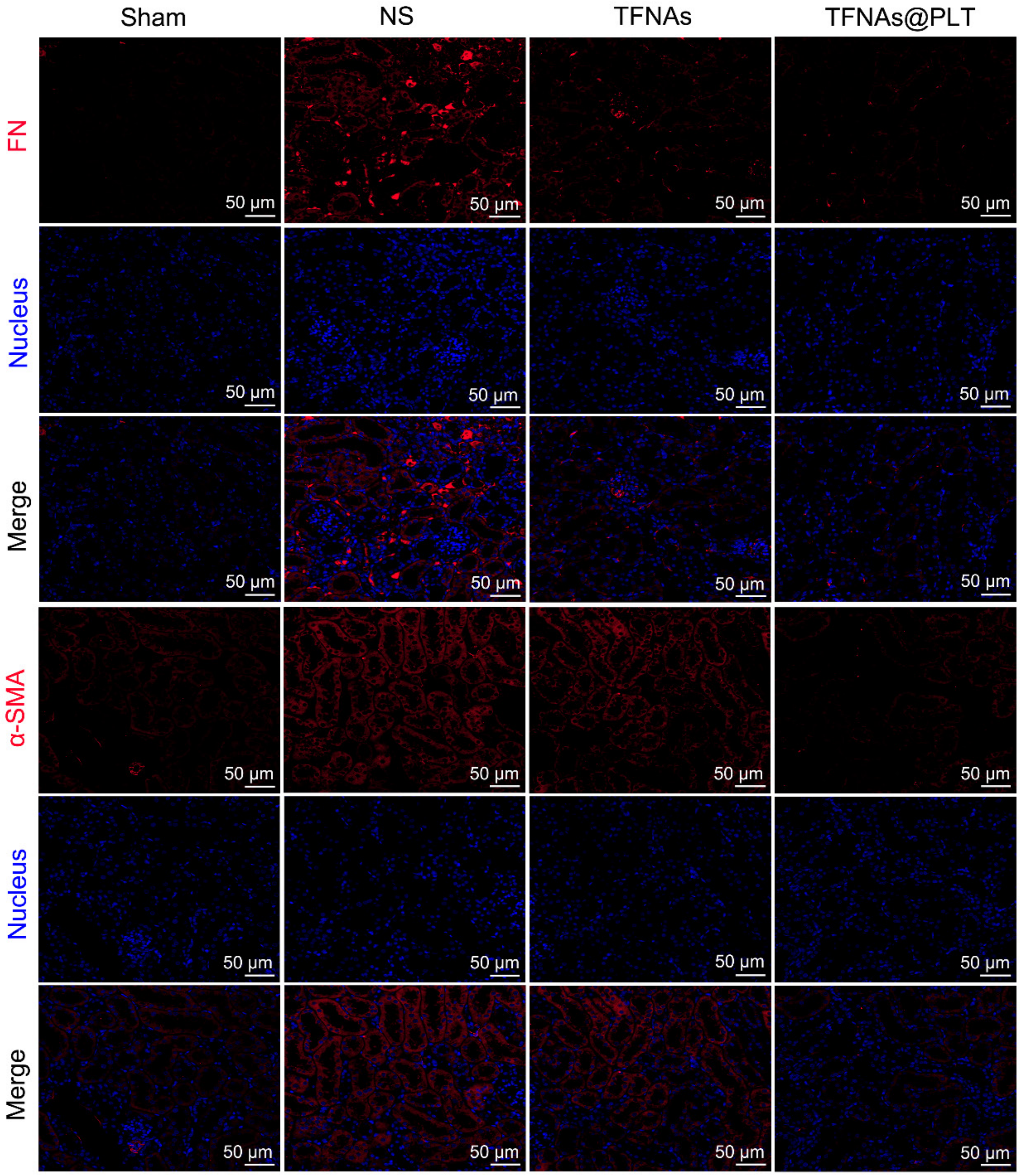

Figure S3: Effects of TFNAs@PLT on key markers of kidney fibrosis. The expression levels of fibronectin (FN) and α-smooth muscle actin (α-SMA) were examined by immunofluorescence. FN and α-SMA were stained red with CY5-conjugated secondary antibody, and the nucleus was stained blue with DAPI.
